# The Making of a Modern Hospital
*Previous articles in this series have appeared in The Hospital of Oct. 7, 14, 21, 28, Nov. 4, 18, Dec. 9 and 30.


**Published:** 1912-02-10

**Authors:** 


### THE MAKING OF A MODERN HOSPITAL.*

### The Ward Itself.—II.

We now come to the general plan of the ward, and here again it will be at once evident that there is no one plan which has commended itself to all hospital experts.

### A Necessary Collaboration.

As a general rule it may be stated that the best hospital wards are those which have been designed by hospital architects working in conjunction with medical men who have made a close study of ward work and who are moreover acquainted with the essentials of hospital construction and have kept themselves abreast of the developments of modern times. Where this co-operation between architect and medical man has not been utilised we sometimes see very curious and remarkable examples of perverted ingenuity, for which sometimes the architect and sometimes the medical man, or the lay committee, is responsible. It cannot be too strongly urged that in the construction of a modern hospital the builder, whoever he is, should consult with the people whose life-work is to be carried on within the walls of the institution. The need for an expert assessor, who is well acquainted with modern hospitals in general, and who knows fully the particular circumstances in each individual case, is becoming increasingly recognised by building committees, but a great deal yet remains to be done before we shall see the principle of co-operation between architect, builder, and medical staff cordially welcomed.

Two recent instances of the excellent results to be obtained by such co-partnership are seen in the new Groningen Hospital, which was built after close consultation between the architect, the engineer, and the medical superintendent, who is himself an authority on hospital management and construction, and in the new surgical clinic at Graz, which is to be opened next year and which is the joint work of the architect and the surgeon-in-charge. As another instance we may cite the excellent gynæcological hospital at Moscow and the new clinics at Gottingen. A still more recent instance is the plan for the new Presbyterian Hospital at New York, which has been evolved by a special commission, consisting of the architects and two of the medical staff, who were specially deputed to visit Europe and study the latest developments in hospital construction. This hospital promises to be one of the most interesting from a constructive point of view in America and we hope to give full details of the designs in a future number.

### A Fault in Building Committees.

The failure to take due regard of essentials in ward construction, which we sometimes meet with in modern hospitals that claim to be up to date, is not always to be ascribed to the architect. Lay committees still arrogate to themselves the right to determine certain points on which the architect in consultation with the medical expert should have the final and determining say. Where that is the case the architect cannot be blamed for the results. He is given a certain task to fulfil and he can only fulfil it to the best of his ability. The wonder is not that we have modern hospitals which are travesties of what they should be but that we have so comparatively few of them. Building committees should follow the example of the managers of the Presbyterian and appoint a consultative committee.

In preceding numbers of The Hospital we have given plans of what may be called "model wards." In order to enable the reader to judge for himself the excellence or otherwise of these individual designs, we may summarise the important points which should be held in view by every hospital architect in constructing a plan of a ward. The ward itself must be easily reached from the various annexes, and must enable the beds to be disposed in a proper manner so that each bed obtains the maximum amount of light and air. For that reason it is unwise to have the wards very long and connected by numerous corridors. Modern opinion is strong on the point that each bed should be between two windows, so that it should have light on both sides, not, as is still so frequently the case, lighted by window light on one side only.

### The Best Shape.

The best form for the ward is that of a right-angled quadrilateral. Various modifications have been attempted, but have not proved much of a success. Thus the Antwerp Civil Hospital possesses circular wards (Stuyvenberg Hospital), the wards of the Saint-Denis hospital are slightly elliptical in shape, while some other institutions have polygonal wards, which like the circular modifications allow a certain amount of even lighting and ventilation. This is nearly all that can be said in their favour; against them stands the disadvantage that such "freak wards" are difficult to keep clean and necessitate modifications in the administration which are not always desirable. In general, therefore, the rectangular modifications have proved the most satisfactory.

The ideal ward contains from ten to twelve beds, this number having been found by experience to be the most convenient for administrative purposes. A minimum air-space of thirty-six cubic mètres per bed must be provided for, and it must be remembered that any excess of height of wall over and above twelve feet is so much dead space which ought not to be taken into account in estimating the cubic air-space per bed. For example, in the Holy Ghost Hospital at Rome, one ward contains no fewer than 250 beds, the official cubic space per bed being given as 110. Nevertheless a visitor to this ward is at once struck by the fact that the air in the room is unwholesomely stuffy, and that the ward is much overcrowded. The official figure is obtained in the usual manner by calculating the

*Previous articles in this series have appeared in The Hospital of Oct. 7, 14, 21, 28, Nov. 4, 18, Dec. 9 and 30.

cubic content of the ward, but the walls are nearly fifty feet high.

With regard to the disposition of the beds, it has already been noted that each bed must be placed between two windows. It is in practice more convenient to have only two rows of beds, each row ranged against the long wall, so that the beds stand at right angles to the long axis of the ward. In some hospitals a double row of beds is provided, but this arrangement, even when overcrowding is avoided, is undesirable for administrative reasons, since all the beds must be easily approachable from all sides. It is advisable to place the beds some distance—generally two feet—away from the wall, and a proper interval of at least six feet should be left between each bed. This allows of a free circulation of air around the bed, and provides ample space for the locker and table if necessary.

### The Ward Walls.

The walls of the ward should be even and smooth, all projecting corners carefully rounded, and the surface painted with some easily cleaned and attractively coloured but not too bright wall-paint, both as a means of protection and to add to the appearance of the room. The ceiling should be plainly protected in the same manner. The doors and windows should be according to the most approved models, the latter made to open outwards, with large single panes of glass, and, where the temperature is cold, provided with double panes. In a later article we will deal with these matters in greater detail. All that need be said at present is that it is of the utmost importance that provision should be made for ample window lighting. Hitherto it has been considered sufficient if the architect allows from two to three square mètres of surface lighting per bed, but modern authorities are inclined, and rightly, to insist on the latter figure as a minimum. The heating and ventilation are more conveniently discussed in a separate article.

An important point for the architect to consider is the disposition of the annexes. We have already stated what these annexes are. Formerly it was deemed sufficient to provide for them in a stepmotherly manner; nowadays, however, it is generally agreed that the surface area of the annexes as a whole must be at least equal to the area of the ward itself. In some hospitals the surface area of the annexes is even greater than that of the ward, but this is needlessly extravagant, and, since the ideal of perfection in hospital construction is simplicity, such amplification is to be deprecated.

### Annexes.

Taking the annexes in detail, it may be pointed out that the most common fault to be found in modern wards is the disposal of the lavatory accommodation, both that for the patients and that for the attendants. In some wards the water-closets are ranged close to the bathroom, at a considerable distance from the beds or from the day-room when there is one. When it is remembered that the water-closets are used by patients who can leave their beds, while the baths are used by all patients, some of whom may have to be carried into the bathroom, it is clear that another disposition is preferable. Much stress must be laid on the desirability of having the lavatory annexes properly ventilated and private, both at the patients' and at the attendants' end. Accessory annexes for the disposal of urine, septic matter, and foul water generally, must be provided, and these must be isolated from the main lavatory, and not, as is usually the case, built alongside. Indeed the proper disposition of the lavatory annexes is a matter which demands the most careful consideration and often presents many difficulties which will have to be overcome.

